# Exceptional Hyperthyroidism and a Role for both Major Histocompatibility Class I and Class II Genes in a Murine Model of Graves' Disease

**DOI:** 10.1371/journal.pone.0021378

**Published:** 2011-06-27

**Authors:** Sandra M. McLachlan, Holly A. Aliesky, Chun-Rong Chen, Robert W. Williams, Basil Rapoport

**Affiliations:** 1 Thyroid Autoimmunity Unit, Cedars-Sinai Research Institute and UCLA School of Medicine, Los Angeles, California, United States of America; 2 Department of Anatomy and Neurobiology, University of Tennessee Health Science Center, Memphis, Tennessee, United States of America; Cardiff University, United Kingdom

## Abstract

Autoimmune hyperthyroidism, Graves' disease, can be induced by immunizing susceptible strains of mice with adenovirus encoding the human thyrotropin receptor (TSHR) or its A-subunit. Studies in two small families of recombinant inbred strains showed that susceptibility to developing TSHR antibodies (measured by TSH binding inhibition, TBI) was linked to the MHC region whereas genes on different chromosomes contributed to hyperthyroidism. We have now investigated TSHR antibody production and hyperthyroidism induced by TSHR A-subunit adenovirus immunization of a larger family of strains (26 of the AXB and BXA strains). Analysis of the combined AXB and BXA families provided unexpected insight into several aspects of Graves' disease. First, extreme thyroid hyperplasia and hyperthyroidism in one remarkable strain, BXA13, reflected an inability to generate non-functional TSHR antibodies measured by ELISA. Although neutral TSHR antibodies have been detected in Graves' sera, pathogenic, functional TSHR antibodies in Graves' patients are undetectable by ELISA. Therefore, this strain immunized with A-subunit-adenovirus that generates only functional TSHR antibodies may provide an improved model for studies of induced Graves' disease. Second, our combined analysis of linkage data from this and previous work strengthens the evidence that gene variants in the immunoglobulin heavy chain V region contribute to generating thyroid stimulating antibodies. Third, a broad region that encompasses the MHC region on mouse chomosome 17 is linked to the development of TSHR antibodies (measured by TBI). Most importantly, unlike other strains, TBI linkage in the AXB and BXA families to MHC class I and class II genes provides an explanation for the unresolved class I/class II difference in humans.

## Introduction

Susceptibility to Graves' disease has long been associated with genes of the major histocompatibility complex (MHC; HLA in humans)(reviewed in [1]). Hyperthyroidism in Graves' patients is caused by autoantibodies to the thyrotropin receptor (TSHR) that mimic the stimulatory activities of the ligand (TSH) on the thyroid gland (reviewed in [2]). Associations between particular autoimmune diseases and MHC amino acid sequences [3] likely reflect the ability of the MHC class II binding pocket to accommodate peptides that stimulate autoreactive T cells, as shown recently for thyroglobulin [4]. A similar mechanism would be expected to play a role in MHC class II binding for peptides of the thyrotropin receptor (TSHR), the autoantigen in Graves' disease. Consistent with this possibility, MHC class II region genes were most tightly linked to susceptibility in a genome-wide association scan in thyroid autoimmune disease, which included an expanded cohort of Graves' patients [5]. However, numerous early studies reported associations between class I (B8) in addition to class II (DR3) genes (for example [6]). Besides MHC class I and II, a recent study found “a novel and major association of HLA-C in Graves' disease that eclipses the classical HLA-DRB1 effect” [7].

Graves' disease can be induced in mice by injecting cells expressing the human TSHR or immunization with the human TSHR DNA in plasmid or adenovirus vectors (reviewed in [8]). Surprisingly, in several mouse models of induced Graves' disease, initial studies suggested that MHC genes were less important susceptibility factors than non-MHC genes (for example [9]); reviewed in [10]). However, later linkage studies with recombinant inbred (RI) strains - essentially families of related strains of mice - provided an answer to this apparent discrepancy in terms of the MHC gene contribution to human versus murine Graves' disease susceptibility. In two RI families (CXB and BXH), development of TSHR antibodies was linked to loci in the MHC region whereas genes on different chromosomes were linked to hyperthyroidism [11], [12]. These studies in CXB and BXH strains demonstrated a role for MHC region genes in controlling the generation of TSHR antibodies, at least as measured by inhibition of TSH binding to its receptor (TBI). However, more detailed mapping of genes within the MHC region was not performed for these two small RI families because each only contained 13 strains.

The AXB and BXA families of strains (here abbreviated AXBXA) were derived from parental strains A and C57BL/6 (B6). B6 mice are also one parental strain in the CXB and BXH sets [13]. Mice of the B6 strain are “good antibody responders” to immunization with adenovirus expressing the TSHR or its A-subunit but rarely develop hyperthyroidism [14], [15]. Mice of the A strain have not previously been examined for their response to TSHR immunization. However, A strain mice have been investigated for antibody induction to a variety of antigens including phosphorylcholine [16], staphylococcal nuclease IV [17] and hen-egg lysozyme [18] and for their responses to infectious organisms such as *Plasmodium falciparum* [19].

The A strain is particularly interesting in terms of its MHC genes compared with those in B6, C3H/He and BALB/c mice. The latter two (C3H/He and BALB/c) are the non-B6 parents of the BXH and CXB families, respectively. The MHC class II genes of A mice are of the *k*-haplotype, as in C3H/He mice, in contrast to the *d*- and *b*-haplotypes of B6 and BALB/c mice. Moreover, the MHC class I genes of A strain mice are a mixture of *k*- and *d*-haplotypes [20]. For these reasons, and because the collected AXBXA set is significantly larger (26 strains) than the previously studied families, we investigated the outcome of immunizing AXBXA strains with adenovirus expressing the TSHR A-subunit. Our findings provide several unexpected and intriguing results, particularly in relation to the contribution of MHC region genes to the generation of TSHR antibodies.

## Results

### TSHR antibodies and hyperthyroidism induced in A and B6 mice and their F1 offspring

**Figure 1:**
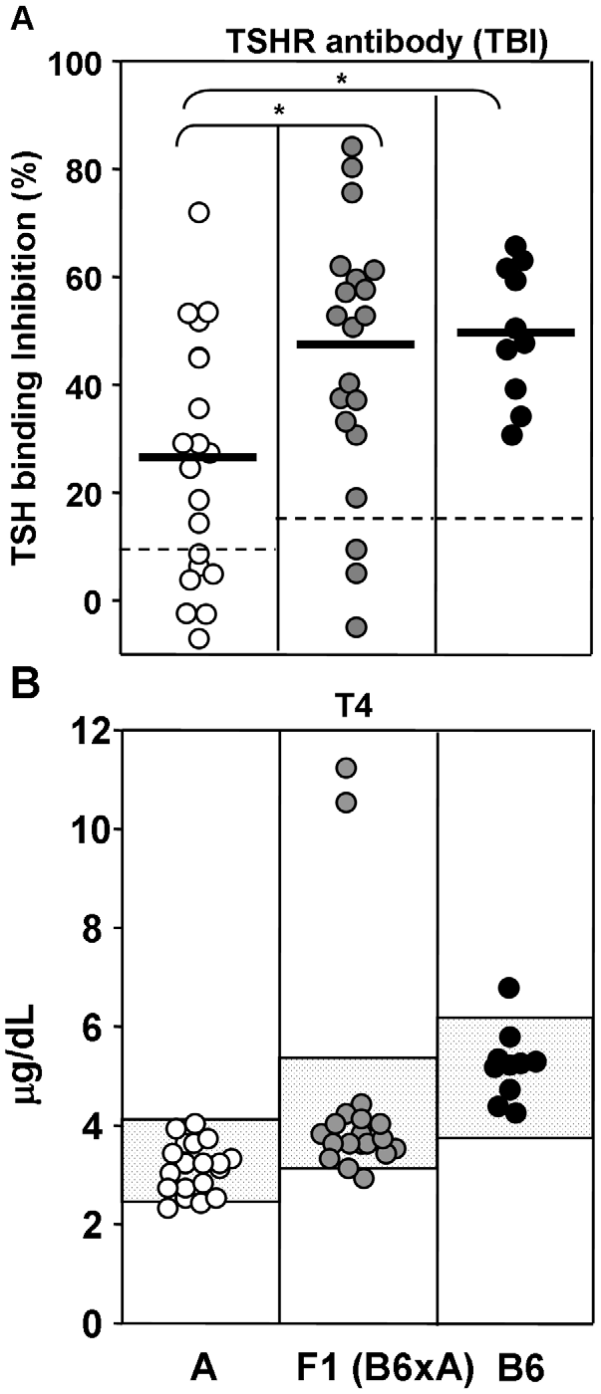
TSHR antibodies measured by TSH binding inhibition (TBI) and serum T4 in A/J, B6AF1 and B6 mice immunized three times with TSHR A-subunit-adenovirus; B6 data from Chen et al [**15**]. Values are shown for individual mice (white, A/J; grey, B6XA F1; black, B6). A) TBI (% inhibition of labeled TSH binding to its receptor). Broad black bars indicate the mean TBI values; horizontal broken lines: mean + 2SD for control-adenovirus immunized mice. *, values significantly greater than for B6 mice (ANOVA; Holme -Sidak method, p<0.05). B) T4 (µg/dL). Speckled box: mean + 2SD for Con-Ad immunized mice of each strain.

Mice of the A/J strain (referred to as “A”) immunized three times with TSHR A-subunit-Ad developed significantly lower TSHR antibody levels (measured by TSH binding inhibition, TBI) than either B6 mice or the F1 hybrids (B6XAF1) between the two parental strains (Fig. 1A; *p* <0.05, ANOVA). In terms of thyroid function, no A strain mice and only one B6 mouse had elevated serum T4 compared with Con-Ad immunized animals of the same strain. However, two of 20 B6XAF1 mice immunized with TSHR A-subunit-Ad were clearly hyperthyroid (Fig. 1B).

### Response of recombinant AXBXA strains to A-subunit adenovirus immunization

**Figure 2:**
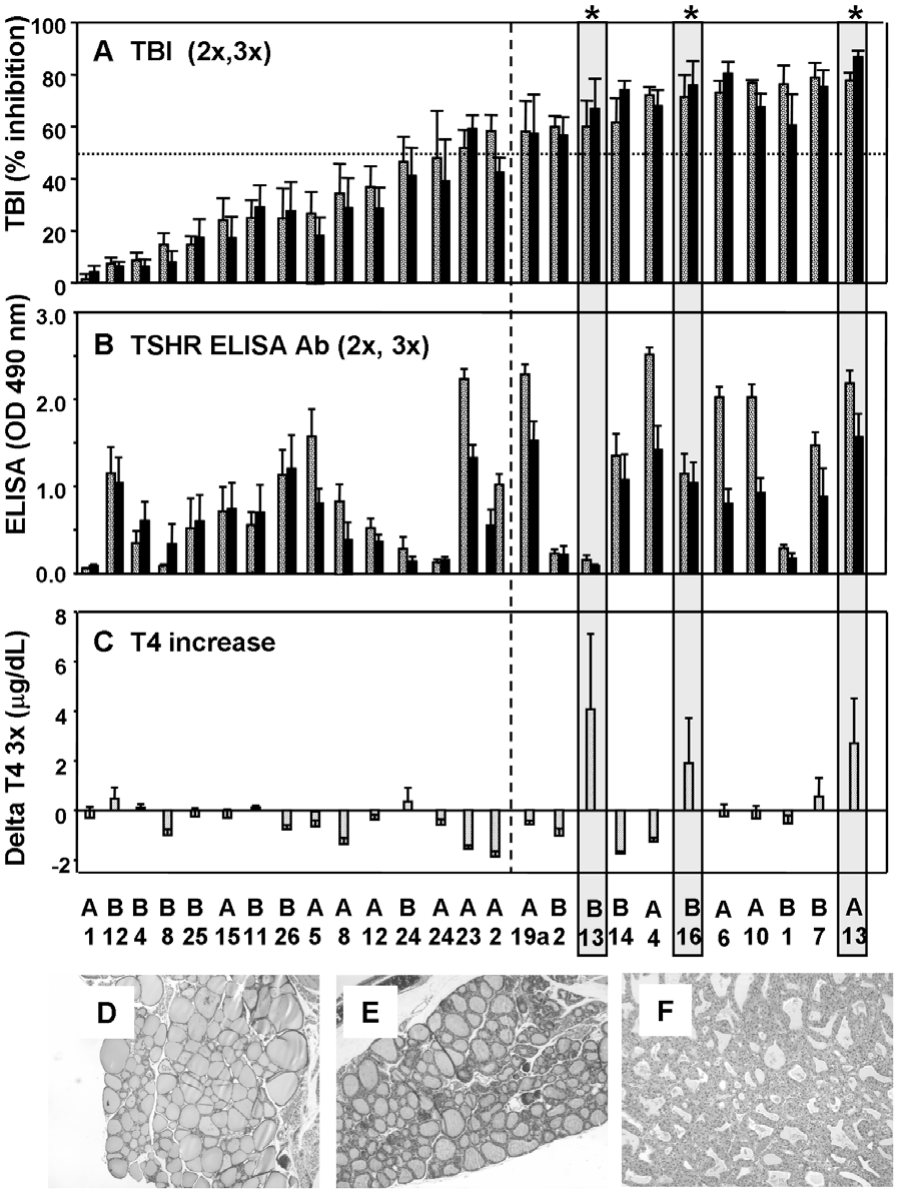
TSHR antibodies, T4 and thyroid histology in 26 AXBXA strains immunized three times with TSHR A-sub-Ad. Data are ranked from left to right according to TBI activity (after the third immunization) from the strain with the lowest TBI (AXB1, abbreviated A1) to the strain with the highest TBI activity (AXB13, A13). Grey boxed areas with an asterisk link panels A,B and C for the strains with the greatest increase (delta) in T4, namely BXA13, BXA16 and AXB13 (B13, B16 and A13, respectively). A) TBI (inhibition of labeled TSH binding to its receptor) and B) TSHR antibody measured by ELISA (OD 490 m). Bar graphs represent the mean +SEM (5–6 mice/strain) after 2 immunizations (2x, speckled bars) or 3 immunizations (3x, solid bars). The vertical dashed line indicates the cut-off between strains with less than 50% TBI (to the left) and greater than 50% TBI (right of the line). C) T4 increase (delta T4 3x). Data shown as bars graphs for the mean + SEM. D, E, F) Representative thyroid histology (magnification 10x) for euthyroid mouse (D), hyperthyroid mouse with T4 10 µg/dL (E) and extremely hyperthyroid mouse with T4 ∼20 µg/dL (F, as in BXA13 strains).

After immunization with A-subunit-Ad, 15 of 26 AXBXA strains developed low TBI levels, from almost negative to <50% inhibition of TSH binding (Fig. 2A; strains ranked left to right for TBI, lowest to highest). The TBI response in the other 11 strains was more robust (>50%). TBI values were similar after two or three immunizations (Fig. 2A, speckled versus solid bars, respectively). In contrast, when measured by ELISA, TSHR antibody levels for some strains were lower after the third versus the second immunization (Fig. 2B). Moreover, the rank order for TSHR antibodies measured by ELISA differed from that for TBI. Focusing on the strains with strong TBI responses, TSHR antibodies measured by ELISA were disproportionately low, indeed almost undetectable, in strains BXA2, BXA13 and BXA1 (labeled B2, B13 and B1) (Fig. 2B versus 2A).

Turning to thyroid function, T4 levels were virtually unchanged in AXBXA strains after two immunizations (data not shown). However, after the third immunization, serum T4 was markedly increased in BXA13, BXA16 and AXB13 strains (labeled B13, B16 and A13), as reflected in the increase above pre-immunization levels (“delta” T4 values; Fig. 2C). Indeed, one BXA13 mouse had the highest absolute T4 level (22 µg/dL) that we have ever observed over 8 years in any mouse strain. Thyroid histology confirmed that thyroid hyperplasia in BXA13 far exceeded that routinely observed for other hyperthyroid AXBXA strains (Fig. 2F versus 2E; euthyroid tissue in Fig. 2D) or even hyperthyroid mice of the susceptible BALB/c strain (for example [21]. In BXA13 strains (which had the largest increase in serum T4), there was a striking discordance between the very strong serum TBI activities (∼70%; Fig. 2A) and the virtual absence of TSHR antibodies detectable by ELISA (Fig. 2B).

### TSAb activity

**Figure 3:**
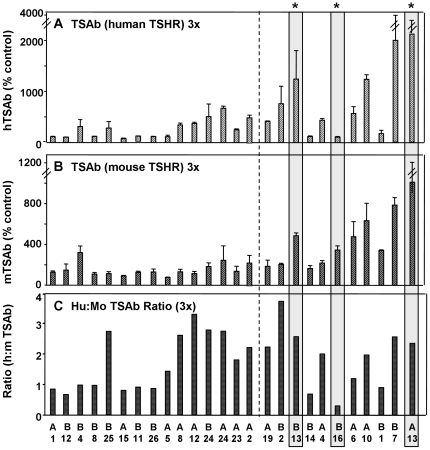
Thyroid-stimulating antibodies (TSAb) in AXBXA strains after 3 immunization with A-sub-Ad. Data are shown for TSAb specific for the human-TSHR (A), the mouse-TSHR (B), and as the ratio of human: mouse TSAb (C). Error bars represent SEM (A and B). As in Fig. 2, the grey boxed areas with an asterisk link panels A), B) and C) for the three most hyperthyroid strains, namely BXA13, BXA16 and AXB13 (B13, B16 and A13, respectively); also, the vertical dashed line indicates the “cut-off” for strains with less than 50% TBI.

Thyroid stimulating antibody (TSAb) activity was measured in AXBXA strains after the third A-subunit-Ad immunization. Separate assays were performed using CHO cells stably expressing the human-TSHR or the mouse-TSHR. The rank order for TSAb values corresponded approximately to that for TBI. Strains with the highest TSAb values, specific for the human- or the mouse-TSHR, generally had high TBI levels (Fig. 3A and B versus Fig. 2A). Another measure of TSAb activity, the ratio of human:mouse-TSHR responsivity (Fig. 2C), indicated preferential recognition of the human versus the mouse-TSHR consistent with the immunogen being the human-TSHR A-subunit.

### Linkage analysis in AXBXA mice

Quantitative trait loci (QTL) were mapped using the AXBXA strain database files [13], [22] for the following parameters: TSHR antibodies measured by TSH binding inhibition (TBI), ELISA, TSAb activity (specific for the human-TSHR, the mouse-TSHR, or the human:mouse TSAb ratio); T4 levels and the difference “delta”) between T4 after immunization and baseline.

The most striking linkage was between TBI and a locus on Chr 17 (twin peaks at ∼33 and ∼37 Mb) with likelihood ratio statistic (LRS) values of 24.2 (after two immunizations) and 32.5 (after three immunizations)(Table 1). The latter LRS value (corresponding to LOD score 7.05) greatly exceeds the LRS cut-off of 20–25 usually required to reach genome-wide significance of *p*<0.05. TSHR antibodies measured by ELISA (after two and three immunizations) were linked to loci on Chr 1, with a higher LRS value after the second than after the third immunization (Table 1), likely associated with higher ELISA antibody values after two than after three adenovirus injections (Fig. 2B). TSAb specific for the mouse-TSHR (m-TSAb) was linked to the same loci on Chr 17 region as TBI (Table 1). However, TSAb specific for the human-TSHR (h-TSAb) and the ratio of TSAb activities with the human or mouse TSHR (h:m-TSAb) were linked to a locus on the distal end of Chr 12. In terms of thyroid function, the absolute serum T4 level and the increase in T4 (“delta T4”) after three immunizations were linked to loci on Chr 16.

**Table 1:** Chromosomal linkage for TSHR antibodies and thyroid function in AXBXA RI mice immunized with TSHR A-subunit-Adenovirus.

Trait	LRS	LOD^a^	Chr	Locus	Mb	h-Chr^b^
**TSHR Antibodies**						
TBI (3x)	**32.52**	**7.05**	**17**	rs3672987	33.247	19
				rs13482968	37.269	
ELISA (2x)	20.19	4.38	**1**	rs4222856	180.251	1
				CEL-1_178482360	180.396	
ELISA (3x)	15.08	3.27	**1**	D1Mit356	174.819	FcR-like6^d^
				rs4136041	177.367	
m TSAb (3x)^c^	14.10	3.06	**17**	rs3672987	33.247	19
				rs13482968	37.269	
h TSAb (3x) c	15.11	3.28	**12**	D12Nds2	115.135	14
				rs3686531	120.968	
**Thyroid Function**						
T4 (3x)	14.52	3.15	**16**	rs4192837	60.329	3
				rs4195972	64.435	
Delta T4 (3x)	10.45	2.27	**16**	rs4202837	73.290	3
				rs4203607	73.919	

Loci and their chromosomal locations (megabases, Mb) are given for traits with the highest LRS (Likelihood Ratio Statistic) scores after two or three (2x or 3x) immunizations; significant linkage in bold.

a LOD scores: calculated from LRS/4.61.

b Information on the corresponding human (h) Chr or likely Chr is included.

c Linkage analysis performed using SEM;

d Fc receptor-like 6 (174.528328 Mb). Note: Lower LRS values:- TBI (2x): LRS 24.21 (33.2472, 37.2686 Mb);TSAb h:m ratio (3x): LRS 14.85 (115.135, 120.968 Mb).

### Linkage for TSHR antibodies in AXBXA, CXB and BXH sets compared with previous reports on AXBXA responses to other immune challlenges

We compared linkage data for induced TSHR antibodies in the AXBXA set with our previous findings in CXB and BXH sets [11], [12], [23] and with information on the responses in the AXBXA set to other immune challenges [20], [24]:-

**Figure 4:**
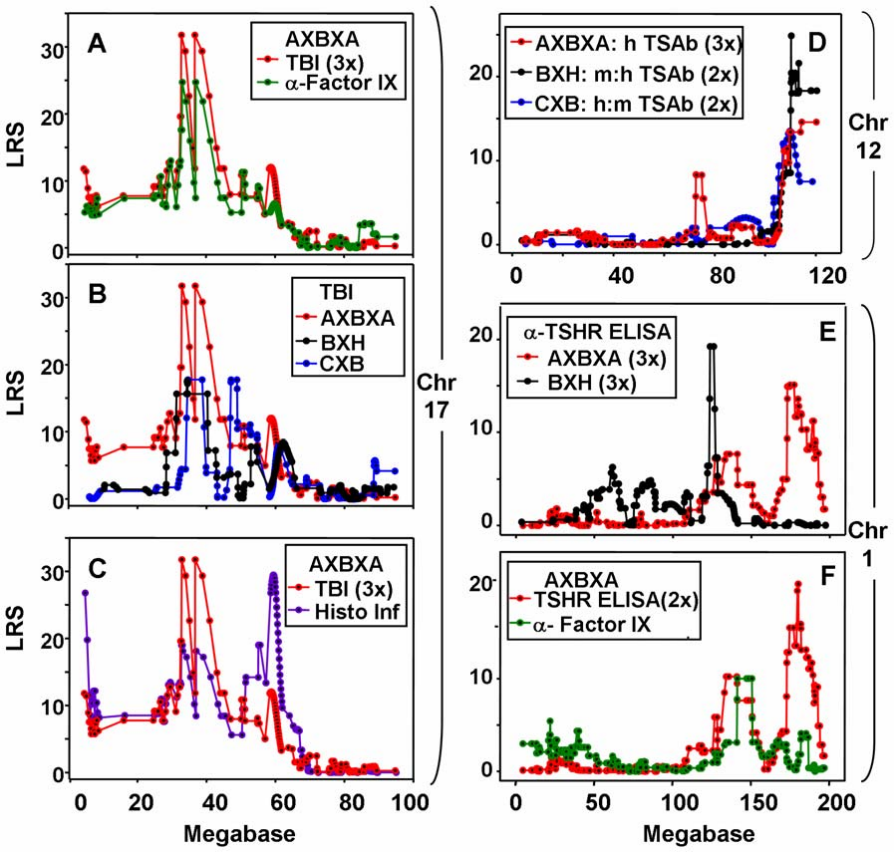
Chromosomal linkage in AXBXA, CXB and BXH sets for induced antibodies TSHR antibodies [present study; [**11]**, [12****] as well as other immune response in AXBXA strains: antibodies to human factor IX induced using adenovirus [**20**] and spleen weight in *Histoplasma capsulatum* infected mice [**24**]. For each panel, chromosomal location is on the X-axis (Mb) and LRS values are on the Y-axis. Left panels: Chr 17 linkage for A) TBI 2x and anti-factor IX in AXBXA strains; B) TSHR antibodies measured by TBI after 2 immunizations in AXBXA, BXH and CXB strains; C) TBI (2x) and *H. capsulatum* infection (spleen weight) in AXBXA strains. Right panels: D) Chr 12 linkage for hTSAb in AXBXA, m:h TSAb in BXH and h:m TSAb in CXB strains; E) Chr 1 linkage for antibodies measured by ELISA in AXBXA and BXH strains; F) anti-TSHR by ELISA 2x versus anti-factor IX in AXBXA strains.

- The most notable similarity is the near identical linkage between TBI and the Chr 17 locus (present study) and induced antibodies to human Factor IX (anti-Factor IX) [20](Fig. 4A). In addition, the linkage on Chr 17 for TBI overlapped in all three RI sets. However, for BXH and CXB mice, LRS values were lower, broader and spanned the two AXBXA peaks (Fig. 4B). Variability in the response of AXBXA strains to infection with *Histoplasma capsulatum* (measured as spleen weight) was also linked to the Chr 17 locus but the major peak was distal to the peaks for TBI and anti-factor IX (Fig. 4C versus Fig. A and B).
- TSAb activities, specific for the human-TSHR, or the h:m-TSAb ratio, in AXBXA, BXH and CXB sets were all linked to loci on the distal end of Chr 12 (Fig. 4D).
- Linkage to Chr 1 loci for TSHR ELISA antibodies in AXBXA mouse strains overlapped to a minor extent with the values for BXH strains (Fig. 4E) or versus anti-factor IX antibodies in AXBXA strains (Fig. 4F).

### Contribution of parental B6 alleles versus non-B6 alleles

Information on the contribution of parental alleles is provided by the additive effects for linkage. Positive additive effects indicate the contribution from *A*-strain alleles (A strain mice) whereas negative additive effects indicate the contribution from *B*-alleles (B6 strain mice)(see Methods).

**Figure 5:**
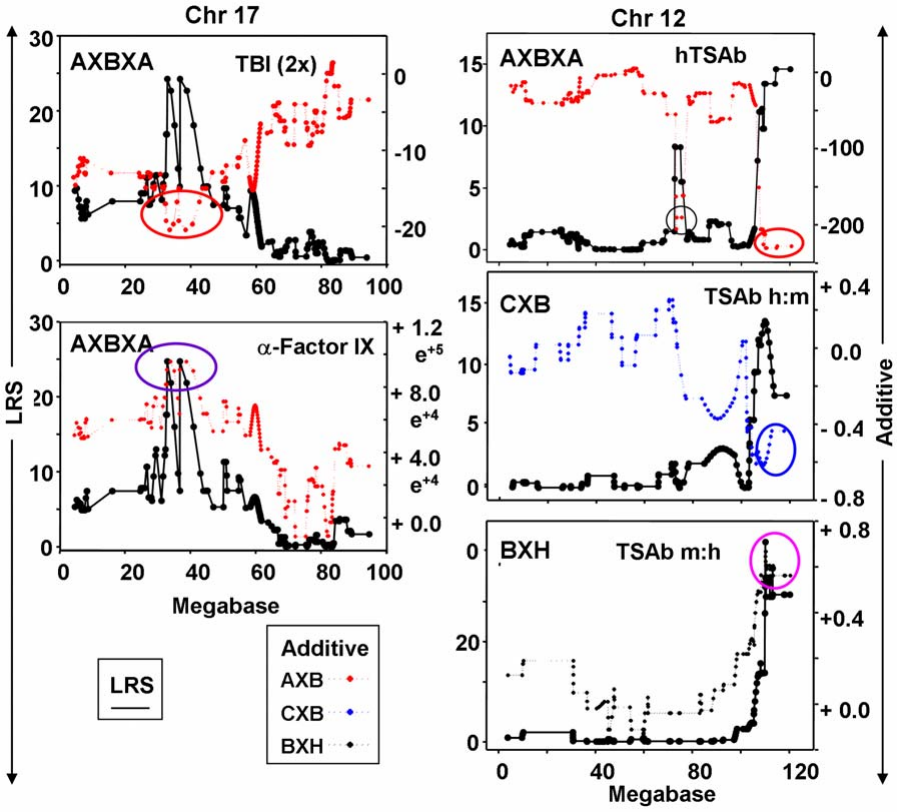
Influence of parental genes (B6 or non-B6) on linkage between TBI and Chr 17 (left panels) and between TSAb and Chr 12 (right panels) in AXBXA, CXB and BXH sets. Also included are data for antibodies to Factor IX in AXBXA mice. LRS values are plotted together with the “Additive effect” which is defined as:- half the difference in the mean phenotype of all cases homozygous for one parental allele at this marker minus the mean of all cases homozygous for the other parental allele at this marker. For AXBXA strains: positive additive effects indicate that A alleles increase trait values; negative additive effects indicate that B6 alleles (red ellipse) increase trait values. Chromosomal location is on the X-axis (Mb); LRS values on the left Y-axis; additive values on the right Y-axis. Elipses highlight Chr locations for peak LRS values. Left: TBI activity in AXBXA mice (upper panel) and anti-human factor IX (lower panel, from [20]. Additive effects:- positive, A alleles (purple ellipse); negative, B6 alleles (red ellipse). Right panels: Upper right, TSAb specific for the human TSHR in AXBXA mice; middle right, TSAb human: mouse (h:m) ratio in CXB strains (from [23]; lower right, TSAb mouse: human (m:h) ratio in BXH mice (from [12]. Additive effects:- positive, C3H/He allelles (blue ellipse) or BALB/c (pink ellipse); negative, B6 alleles (red ellipse).

- TBI:- the **negative** additive effects (−20) indicate the contribution from B6 variant genes (Fig. 5, top left; red ellipse). In contrast, the strong **positive** additive effects (>8000) demonstrate that anti-Factor IX levels are increased by alleles inherited from the A-strain (Fig. 5, bottom left, purple ellipse).
- TSAb, in AXBXA, BXH and CXB sets, TSAb measurements involving the *human*-TSHR were linked to Chr 12 loci. For AXBXA strains, the negative additive effects indicate the contribution of *B* alleles (red ellipse. Fig. 5 top right); in CXB and BXH strains, the positive additive effects reflect the contribution of alleles from BALB/c and C3H/He strains (blue and pink ellipses, respectively; Fig. 5, right middle and bottom panels). Incidentally, in the AXBXA set, TSAb specific for the *mouse*-TSHR was linked to the same Chr 17 locus as TBI (Table 1) and, as for TBI, *B* alleles increased this trait (data not shown).

LRS values for TSAb activities in the three RI sets individually are relatively low (Table 1; [23]. However, combined linkage analysis increased the LOD scores for linkage between TSAb (specific for the human-TSHR or the human:mouse-TSAb ratio) and Chr 12 (Table 2).

**Table 2:** Combined linkage for Chr 12 loci for TSHR antibodies measured by TSAb in AXBXA (present study), BXH and CXB sets [23].

Trait	Individual LOD	Interval (Mb)	X^2^	p	Com LOD^b^
**TSAb** a					
AXBXA (3x) h	3.28				
BXH (3x) h	4.05	113.270–114.345^c^	48.32	1.01923E-08	**7.99**
CXB (2x) m	3.16				
**TSAb ratio** (h:m)					
AXBXA (3x)	3.23				
BXH (3x)	3.14	113.595–117.869^d^	36.80	1.93028E-06	**5.71**
CXB (2x)	1.62				

a TSAb was assayed using cells expressing human (h) or mouse (m) TSHR. Mice received two or three immunizations (2x or 3x, respectively).

b Comb LOD: combined LOD score.

c 113.27–114.345 Mb: 14/40 (35%) IgH V region genes.

d 115.135–117.869 Mb: 14/26 (54%) IgH V region genes.

### Genes on Chr 17 linked to TBI activity

**Figure 6:**
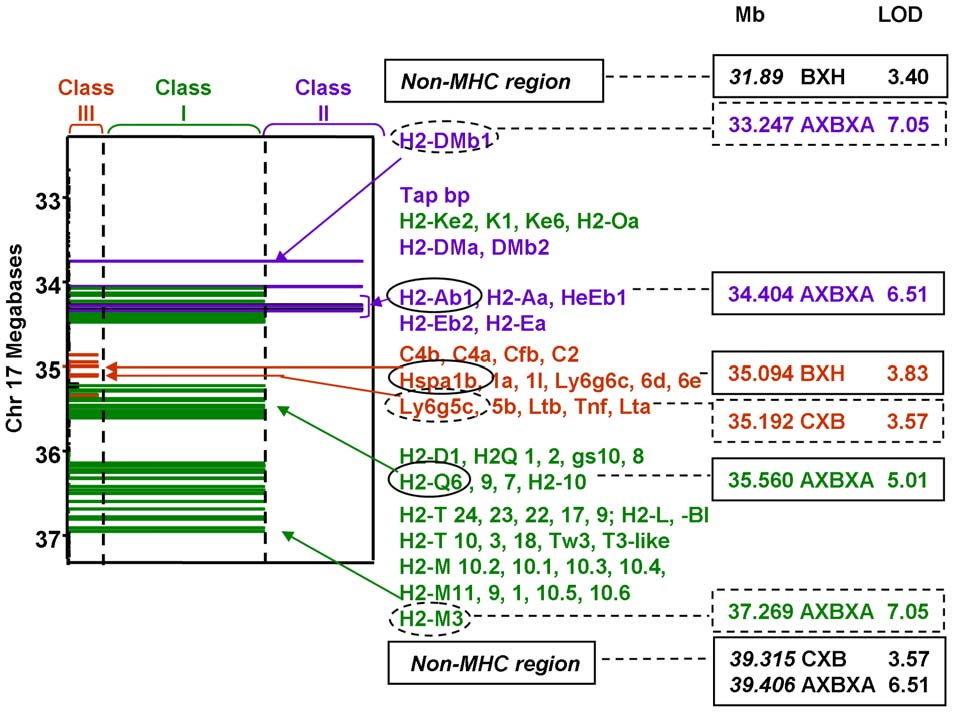
MHC region genes on mouse Chr 17 in relation to TBI linkage in AXBXA, CXB and BXH RI sets. Genes are clustered and assigned arbitary colors and bar lengths to distinguish between the following:- *Green*: MHC Class I genes (K, -O,; -D1 and -Q; H2 -T, -L and -M; *Purple*, MHC Class II genes: H2-Ab1, Aa, Eb1, Eb2, Ea, as well as Dm and Tap; *Brown*: MHC class III genes complement, tumor necrosis factor, lymphotoxin, heat shock proteins (C, Tnf, Ltn, hsp) and 5 groups of lymphocyte antigen-6 (Ly-6): Ly6g6c,d,e and Ly6g5c,and b; *Black*: Non-MHC genes. LOD scores are for RI mice immunized 3x (AXBXA) or 2x (BXH and CXB). Solid elipses: genes at or very close to loci for maximum LOD scores; dashed elipses: closest genes (insufficient markers for precise localization).

The location of class I, II and III MHC genes is illustrated schematically (Fig. 6). Apart from upstream DMb1, MHC class II genes (*Ab1, Aa, Eb1, Eb2, Ea, Dma and DMb2*) lie between class I genes H2-K and the class III genes which include complement, tumor necrosis factor, lymphotoxin, lymphocyte antigen 6 and heat shock proteins (*C,Tnf, Ltn, Ly-6* and *Hsp*). The distal group of MHC genes encode class I molecules (D, M and T).

The strongest TBI linkages in the AXBXA set (LOD >7) are to the MHC class II gene *DMb1* and to a class I gene, tentatively assigned as *H2-M3* (Fig 6). In addition, TBI in AXBXA strains is linked to the class II gene *H2-Ab1* and the class I gene *H2-Q6*. AXBXA LOD scores exceed those for the BXH and CXB sets (3.83 and 3.57, respectively). In BXH strains, TBI is linked to the class III gene *Hspa1* and tentatively linked in CXB strains to *Lysg5c.* Some linkages between TBI and Chr 17 lie outside the MHC region (with no known candidate genes): upstream (BXH set) and downstream of MHC genes (CXB, AXBXA)(Fig. 6, Table 2).

## Discussion

Recombinant inbred AXBXA strains immunized with TSHR A-subunit adenovirus provided insight into two aspects of Graves' disease. First, we observed more severe hyperthyroidism in one of these strains than we previously observed in any other inbred strain. Second, our data are the first to implicate MHC class I genes as contributing to the variability in the TSHR antibody response in an induced mouse model of Graves' disease. Neither of these outcomes was anticipated from studying the parental strains and the F1 obtained by crossing A to B6 strains. Briefly, TSHR antibody responses (measured as TBI) were low in A-strain mice and high in the F1, as in B6 mice, demonstrating that high responder B6 parental genes dominate the low responder A-strain parental variants. Moreover, although uncommon, some F1 mice became hyperthyroid. Consequently, it seemed likely that studying the relatively large AXBXA set (26 members) would contribute to understanding the genetic susceptibility to induced Graves' disease. However, the overall impact was far greater than we had expected.

Very severe hyperthyroidism associated with extreme thyroid follicular cell hyperplasia (Fig. 2F) was observed in BXA13 mice. We offer what we believe to be a likely explanation for this phenomenon. TSHR antibody assays for inhibition of TSH binding (TBI) and TSHR activation (TSAb) measure pathogenic (‘functional’) antibodies. Immunization of BALB/c and B6 mice with A-subunit adenovirus induces *both* functional TSHR antibodies detectable by TBI and TSAb as well as antibodies measured by ELISA (for example [15]). Pathogenic (or ‘functional’) TSHR antibodies, including the human monoclonal stimulating antibody M22 [25], have little or no recognition of the purified, recombinant TSHR A-subunit protein coated on an ELISA plate. Despite high TBI values, BXA13 mice were virtually negative for TSHR-ELISA antibodies.

It should be noted that neutral TSHR antibodies have been detected in Graves' sera by flow cytometry [26] or by inhibiting monoclonal antibody binding to ELISA plates coated with peptides corresponding to the TSHR cleavage region, amino acid residues 316–366 [27]. Such neutral antibodies may play a role in exacerbating the autoimmune response in Graves' disease [27]. In contrast, ELISA-type TSHR antibodies induced in mice bind predominantly to the extreme amino-terminus of the TSHR [28]. In fact, the A-subunit used for immunization lacks the cleavage region recognized by most neutral TSHR antibodies [27]. Moreover, as described below, TSHR-ELISA antibodies in mice protect, rather than exacerbate, responses to the TSHR.

Pre-treating BALB/c mice with TSHR A-subunit protein before A-subunit adenovirus immunization attenuated hyperthyroidism by “deviating” the humoral response from pathogenic (TBI and TSAb positive) towards TSHR-ELISA antibodies [29]. Consistent with the extreme degree of hyperthyroidism, the qualitative balance in the BXA13 strain was almost entirely towards functional TSHR antibodies, with minimal ELISA-positive antibodies. Even BALB/c mice, the most susceptible to developing hyperthyroidism in our induced Graves' disease model, develop high levels of TSHR-ELISA antibodies as well as functional TSHR antibodies (for example [21]). Therefore, the BXA13 strain immunized with the TSHR A-subunit may resemble human Graves' patients more closely than do similarly immunized BALB/c mice. Unlike some other autoimmune diseases such as type I diabetes, Graves' disease occurs only in humans and there are no spontaneous animal models of the disease. The importance of our present finding is that the use of BXA13 mice may provide a major enhancement in studying induced Graves' disease.

Our initial goal for investigating mice of the AXBXA set was to expand our studies of the genetic basis for susceptibility to induced hyperthyroidism and TSHR antibodies. In this set, elevated serum T4 was linked to a locus on Chr 16. This linkage was not observed in comparable studies of BXH and CXB strains (Chr 3, 13)[11], [12]. As previously observed [30], linkage of baseline T4 in AXBXA strains (Chr 2)[30] is distinct from linkage in BXH and CXB strains (Chr 1, 11 and 13). Similarly, TSHR antibodies measured by ELISA were linked to Chr 1 but the loci were different from those previously observed in CXB and BXH strains [11], [12]. In contrast, TSAb activity was linked to the same distal region on Chr 12 noted for BXH and CXB strains [23]. Indeed, combined linkage for the three RI sets (AXBXA, CXB and BXH) increased the LOD score from 3.54 in two RI sets [23] to 5.71 in three RI sets over a broad interval (113.595–117.869 Mb). More than half the genes within this interval (54%) encode immunoglobulin heavy chain V region genes. The high frequency of VH genes in this locus suggests that VH gene differences between mouse strains underlie the susceptibility (or lack thereof) to develop antibodies capable of activating the TSHR (as discussed previously [23]).

The most striking observation for the AXBXA set was the very strong linkage between TSHR antibodies measured by TBI and a broadly defined MHC region on Chr 17 with LOD scores rising from 5.25 after two immunizations to 7.05 in mice immunized three times. The same Chr 17 locus was linked in AXBXA strains immunized to develop antibodies to Factor IX [20] and, albeit to a lesser extent, to spleen responses after infection with *Histoplasma capsulatum* [24]. Linkage to Chr 17 is not a general characteristic of AXBXA strains to immunization: the major linkage peak for *Histoplasma* susceptibility is downstream of the MHC region. Moreover, variability in anti-cardiac sacrolemmal antibodies that develop in AXBXA mice after Coxsackie infection is linked to Chr 5 and 7 [31].

Linkage between Chr 17 loci and both TBI and antibodies to human Factor IX raised the following question: because both antibodies were induced using human-adenovirus vectors, do these linkages merely reflect susceptibility to adenovirus? This issue was refuted by information on additive effects: *B*-alleles (**negative** additive effects) increase TBI whereas *A*-alleles (**positive** additive effects) increase anti-Factor IX. Moreover, the major locus for susceptibility to mouse-adenovirus (which resembles human adenoviruses in structure and genome organization) maps to Chr 15 [32], not to Chr 17. Together with the additive effect data, this information supports the conclusion that linkage between TBI and Chr 17, or between anti-Factor IX and the same Chr 17 locus, is unrelated to the adjuvant effects of adenovirus and is specific for the two very different immunogens.

Finally, it was possible to putatively assign MHC genes linked to TBI in AXBXA strains (present study) and in CXB and BXH strains [11], [12]. In the AXBXA set, TBI is strongly linked to *DMb1* (class II) and *H2-Q6* and *H2-M3* (class I) and to a lesser extent to *H2-Ab1* (class II). Although the role of class I antigens is not understood, strong linkage between TBI and *DMb1* suggests that peptide loading to MHC class II antigens plays a critical role in AXBXA responses to A-subunit immunization. Potentially interesting is TBI linkage to the class III genes heat shock protein (BXH set) and a lymphocyte antigen-6 family member (CXB set). The large family of lymphocyte antigen-6 proteins (27 in humans and 37 in mice), have putative immune roles [33]. Heat shock proteins have previously been associated with Graves' disease (for example [34], [35]), although their specific role in thyroid autoimmunity (as distinct from cellular processes in general) is enigmatic [36]. It is intriguing that, at least in some mouse strains, heat shock proteins may be involved in the development of TSHR antibodies.

In conclusion, investigations in AXBXA recombinant inbred mice immunized with TSHR A-subunit adenovirus provided unexpected insight into several aspects of Graves' disease. First, extreme thyroid hyperplasia and hyperthyroidism in one AXBXA strain likely reflects its inability to generate ELISA-type TSHR antibodies ot the type not observed in Graves' patients. For this reason, BXA13 strain immunized with A-subunit-adenovirus may provide the most suitable mouse strain for investigating human Graves' disease. Second, linkage data from AXBXA mice strengthen the case for the contribution of immunoglobulin heavy chain variable region genes to the generation of thyroid stimulating antibodies. Third, genes within and outside the MHC region are linked to the generation of TSHR antibodies (measured by TBI). Moreover, the finding that TBI in AXBXA strains is linked to both class I and class II MHC region genes may provide an explanation for conflicting findings regarding the two classes of these genes in different human populations.

## Materials and Methods

### Immunization of mice with TSHR A-subunit adenovirus

Female mice of the following 26 strains (5 to 8 weeks of age; The Jackson Laboratory, Bar Harbor, Maine) were studied:- A/J and B6AF1J (hereafter referred to as “A” and “B6AF1”); AXB/PgnJ strains 1, 2, 4, 5, 6, 8, 10, 12, 13, 15, 19a (formerly AXB18), 23, 24; BXA/PgnJ strains 1, 2, 4, 7, 8, 11, 12, 13, 14, 16, 24, 25, 26. Adenovirus expressing the human TSHR A-subunit (amino acid residues 1–289, A-subunit-Ad) [21] and control adenovirus lacking an inset (Con-Ad)[37] were propagated in HEK293 cells (American Type Culture Collection, Manassas, VA), purified on CsCl density gradients and viral particle concentration was determined from the absorbance at 260 nm [38].

A and B6AF1 strains were immunized intramuscularly three times with A-subunit-Ad (10^8^ particles per injection)(10–20 mice/strain) or Con-Ad (5 mice/strain) on three occasions at three weekly intervals as described [21]. Data previously obtained for B6 mice immunized in the same way [15] are included for comparison. Baseline T4 values in AXBXA strains were published recently [30]: briefly, before immunization, blood samples were drawn from individual mice in each AXB or BXA strain (5–6 mice/strain); sera were pooled for each strain and analyzed in duplicate for T4. Prior studies in CXB and BXH mice had demonstrated consistency for individual mice in pre-immunization samples [11], [12]. Subsequently, for the current study, AXBXA strains were immunized with A-subunit-Ad (10^8^ particles per injection) three times at three weekly intervals. Blood was drawn one week after the second immunization and mice were euthanized four weeks after the 3^rd^ injection to harvest blood and thyroid glands.

### Assays for TSHR antibodies

TSHR antibodies were investigated using three assays: TSH binding inhibition (TBI), ELISA using TSHR A-subunit protein, and a bioassay for thyroid stimulating antibody (TSAb). TBI was determined using a commercial kit (Kronus, Boise, ID): serum aliquots (25 µl) were incubated with detergent solubilized TSHR; ^125^I-TSH was added and the TSHR-antibody complexes were precipitated with polyethylene glycol. TBI values were calculated from the formula:- [1- (TSH binding in test serum - non-specific binding)/(TSH binding in normal serum - non-specific binding)] X 100.

TSHR antibodies (IgG class) were measured by ELISA as previously described [21]. Briefly, recombinant TSHR A-subunit protein secreted by Chinese Hamster Ovary cells with an amplified transgenome [39] was purified from culture supernatants by affinity chromatography [40]. ELISA wells were coated with A-subunit protein (1 µg/ml) and incubated with test sera (duplicate aliquots, 1∶100 dilution). Antibody binding was detected with horseradish peroxidase-conjugated mouse anti-IgG (Sigma Chemical Co., St. Louis, MO) and the signal was developed with o-phenylenediamine and H_2_O_2_. Data are reported as the optical density (OD) at 490 nm.

TSAb activity specific for the human-TSHR or the mouse-TSHR was assayed as descrribed previously [23]. Briefly, Chinese hamster ovary (CHO cells expressing the human-TSHR (or the mouse-TSHR) were plated in 96 well plates and, when confluent, incubated (60 min, 37°C) with test sera diluted 1∶20 in Ham's F12 supplemented with 10 mM Hepes, pH 7.4, and 1 mM isobutylmethylxanthine. After aspirating the medium, intracellular cAMP was extracted with ethanol, evaporated to dryness and resuspended in Dulbecco's PBS. Samples (20 µl) were assayed using the LANCE cAMP kit (PerkinElmer, Boston MA). TSAb activity was expressed as a percentage of cAMP values attained with sera from control, unimmunized mice.

### Serum thyroxine and thyroid histology

Total thyroxine (T4) was measured in undiluted mouse serum (25 µl) using a radioimmunoassay kit (Diagnostic Products Corporation, Los Angeles, CA). T4 values were computed from standards in the kit and expressed as µg/dL at base-line and after two immunizations (2x) or three immunizations (3x). In addition, to incorporate baseline thyroid function, we calculated the change (delta) after two or three immunizations. Thyroid glands were fixed in buffered formaldehyde (pH 7.4), paraffin-embedded and serial sections were stained with hematoxylin and eosin (Research Animal Diagnostic Laboratory, University of Missouri, Columbia, MO).

### Statistical analyses

Significant differences between responses in different groups were determined by Mann Whitney rank sum test or, when normally distributed, by Student's t test. Multiple comparisons were performed using analysis of variance (ANOVA). Tests were performed using SigmaStat (Jandel Scientific Software, San Rafael, CA).

### Genetic linkage analysis

Putative quantitative trait loci (QTL) involved in traits of the AXBXA set before and after A-subunit-Ad immunization were mapped using the genotype files for AXB/BXA RI strains generated by Williams et al. [13] embedded in GeneNetwork. This genotype file consists of 2446 markers with unique strain distribution patterns (www.genenetwork.org/dbdoc/AXBXAGeno.html). The probability of linkage between our traits and previously mapped genotypes was estimated at ∼1 centiMorgan intervals (∼2 megabase; Mb) along the entire genome, except for the Y chromosome. To establish criteria for suggestive and significant linkage, a permutation test was performed (1000 permutations at 1-centimorgan intervals)[41]. This test compares the peak likelihood ratio statistics (LRS; LRS = LOD×4.6, where LOD is the logarithm of the odds) obtained for the properly ordered data with the distribution of peak LRS scores obtained from 1000 random permutations of the same data. Additive effects were also estimated. In AXBXA strains, a positive additive effect indicates that an A allele increases trait values at a particular locus or marker; a negative additive effect indicates that a B allele increases trait values. The primary phenotype data (10 traits) have been entered into the mouse AXB/BXA Phenotype database on GeneNetwork (www.genenetwork.org) under the trait accession identifiers GN 10158 to 10166, 10172 to 10175 and can be found by searching for the name “McLachlan”.

For some parameters, we combined the LRS values for AXB/BXA strains with our previous findings for CXB or BXH strains [11], [12] to provide linkage data for en entire collection of 52 RI strains that all share a B6 parental strain. Following the combined linkage analysis approach for neuron number in two or more families [42], we calculated the probability associated with a Χ^2^ value equal to: −2 (lnP_AXBXA_ + lnP_CXB_ + lnP_ BXH)_) with 6 degrees of freedom, where lnP_AXBXA,_ lnP_CXB_ and lnP_BXH_ are the natural logarithms of the probabilities derived independently for the three RI families in the same chromosomal interval. Combined linkage data are provided as LOD scores (convertible to LRS values by multiplying by 4.61, as described above). In the combined analyses, “point-wise” *p* values are provided for the single points examined (as opposed to genome-wide tests which examine few hundred points).

## References

[1] Jacobson EM, Tomer Y (2007). The Genetic Basis of Thyroid Autoimmunity.. Thyroid.

[2] Rapoport B, McLachlan SM (2007). The thyrotropin receptor in Graves' disease.. Thyroid.

[3] Todd JA, Acha-Orbea H, Bell JI, Chao N, Fronek Z (1988). A molecular basis for MHC Class II - associated autoimmunity.. Science.

[4] Jacobson EM, Yang H, Menconi F, Wang R, Osman R (2009). Employing a recombinant HLA-DR3 expression system to dissect major histocompatibility complex II-thyroglobulin peptide dynamism: a genetic, biochemical, and reverse immunological perspective.. J Biol Chem.

[5] Burton PR, Clayton DG, Cardon LR, Craddock N, Deloukas P (2007). Nat Genet.

[6] Stenszky V, Kozma L, Balazs C, Rochlitz S, Bear JC (1985). The genetics of Graves' disease: HLA and disease susceptibility.. J Clin Endocrinol Metab.

[7] Simmonds MJ, Howson JM, Heward JM, Carr-Smith J, Franklyn JA (2007). A novel and major association of HLA-C in Graves' disease that eclipses the classical HLA-DRB1 effect.. Hum Mol Genet.

[8] Nagayama Y (2007). Graves' animal models of Graves' hyperthyroidism.. Thyroid.

[9] Nagayama Y, McLachlan SM, Rapoport B, Niwa M (2003). A major role for non-MHC genes, but not for micro-organisms, in a novel model of Graves' hyperthyroidism.. Thyroid.

[10] McLachlan SM, Nagayama Y, Rapoport B (2005). Insight into Graves' hyperthyroidism from animal models.. Endocr Rev.

[11] Aliesky HA, Pichurin PN, Chen CR, Williams RW, Rapoport B (2006). Probing the genetic basis for thyrotropin receptor antibodies and hyperthyroidism in immunized CXB recombinant inbred mice.. Endocrinol.

[12] McLachlan SM, Aliesky HA, Pichurin PN, Chen C-R, Williams RW (2008). Shared and unique susceptibility genes in a mouse model of Graves' disease determined in BXH and CXB recombinant inbred mice.. Endocrinol.

[13] Williams RW, Gu L, Qi S, Lu L (2001). The genetic structure of recombinant inbred mice: high-resolution consensus maps for complex trait analysis.. Genome Biol 2: Research.

[14] Nagayama Y, Kita-Furuyama M, Ando T, Nakao K, Mizuguchi H (2002). A novel murine model of Graves' hyperthyroidism with intramuscular injection of adenovirus expressing the thyrotropin receptor.. J Immunol.

[15] Chen CR, Aliesky H, Pichurin PN, Nagayama Y, McLachlan SM (2004). Susceptibility rather than resistance to hyperthyroidism is dominant in a thyrotropin receptor adenovirus-induced animal model of Graves' disease as revealed by BALB/c-C57BL/6 hybrid mice.. Endocrinol.

[16] Rudikoff S, Claflin JL (1976). Expression of equivalent clonotypes in BALB/c and A/J mice after immunization with phosphorylcholine.. J Exp Med.

[17] Pisetsky DS, Sachs DH (1977). The genetic control of the immune response to staphylococcal nuclease VI. Recombination between genes determining the A/J anti-nuclease idiotypes and the heavy chain allotype locus.. J Exp Med.

[18] Semma M, Sakato N, Fujio H, Amano T (1981). Idiotypic analysis of antibodies to hen egg-white lysozyme (HEL). II. Distribution and frequency of occurrence of idiotypes shared by A/J anti-HEL antibody.. Immunol.

[19] Joshi MB, Gam AA, Boykins RA, Kumar S, Sacci J (2001). Immunogenicity of well-characterized synthetic Plasmodium falciparum multiple antigen peptide conjugates.. Infect Immunol.

[20] Lozier JN, Tayebi N, Zhang P (2005). Mapping of genes that control the antibody response to human factor IX in mice.. Blood.

[21] Chen C-R, Pichurin P, Nagayama Y, Latrofa F, Rapoport B (2003). The thyrotropin receptor autoantigen in Graves' disease is the culprit as well as the victim.. J Clin Invest.

[22] Shifman S, Bell JT, Copley RR, Taylor MS, Williams RW (2006). A high-resolution single nucleotide polymorphism genetic map of the mouse genome.. PLoS Biol.

[23] Rapoport B, Williams RW, Chen C-R, McLachlan SM (2010). Immunoglobulin heavy chain variable region genes contribute to the induction of thyroid stimulating antibodies in recombinant inbred mice.. Genes Immun.

[24] Mayfield JA, Rine J (2007). The genetic basis of variation in susceptibility to infection with Histoplasma capsulatum in the mouse.. Genes Immun.

[25] Sanders J, Allen F, Jeffreys J, Bolton J, Richards T (2005). Characteristics of a monoclonal antibody to the thyrotropin receptor that acts as a powerful thyroid-stimulating autoantibody antagonist.. Thyroid.

[26] Metcalfe R, Jordan N, Watson P, Gullu S, Wiltshire M (2002). Demonstration of immunoglobulin G, A, and E autoantibodies to the human thyrotropin receptor using flow cytometry.. J Clin Endocrinol Metab.

[27] Morshed SA, Ando T, Latif R, Davies TF (2010). Neutral antibodies to the TSH receptor are present in Graves' disease and regulate selective signaling cascades.. Endocrinol.

[28] Schwarz-Lauer L, Pichurin PN, Chen C-R, Nagayama Y, Paras C (2003). The cysteine-rich amino terminus of the thyrotropin receptor is the immunodominant linear antibody epitope in mice immunized using naked DNA or adenovirus vectors.. Endocrinol.

[29] Misharin A, Nagayama Y, Aliesky H, Mizutori Y, Rapoport B (2009). Attenuation of induced hyperthyroidism in mice by pre-treatment with thyrotropin receptor protein: diversion of thyroid stimulating antibody to non-functional antibody induction.. Endocrinol.

[30] McLachlan SM, Lu L, Aliesky HA, Williams RW, Rapoport B (2011). Distinct genetic signatures for variability in total and free serum thyroxine levels in four sets of recombinant inbred mice.. Endocrinol.

[31] Traystman MD, Beisel KW (1991). Genetic control of Coxsackievirus B3-induced heart-specific autoantibodies associated with chronic myocarditis.. Clin Exp Immunol.

[32] Spindler KR, Welton AR, Lim ES, Duvvuru S, Althaus IW (2010). The major locus for mouse adenovirus susceptibility maps to genes of the hematopoietic cell surface-expressed LY6 family.. J Immunol.

[33] Mallya M, Campbell RD, Aguado B (2006). Characterization of the five novel Ly-6 superfamily members encoded in the MHC, and detection of cells expressing their potential ligands.. Protein Sci.

[34] Ratanachaiyavong S, Demaine AG, Campbell RD, McGregor AM (1991). Heat shock protein 70 (HSP70) and complement C4 genotypes in patients with hyperthyroid Graves' disease.. Clin Exp Immunol.

[35] Heufelder AE, Goellner JR, Wenzel BE, Bahn RS (1992). Immunohistochemical detection and localization of a 72-kilodalton heat shock protein in autoimmune thyroid disease.. J Clin Endocrinol Metab.

[36] McGregor AM (1992). Heat shock proteins and autoimmune thyroid disease; too hot to handle?. J Clin Endocrinol Metab.

[37] Chen CR, Aliesky HA, Guo J, Rapoport B, McLachlan SM (2006). Blockade of costimulation between T cells and antigen-presenting cells: an approach to suppress murine Graves' disease induced using thyrotropin receptor-expressing adenovirus.. Thyroid.

[38] Mittereder N, March KL, Trapnell BC (1996). Evaluation of the concentration and bioactivity of adenovirus vectors for gene therapy.. J Virol.

[39] Chazenbalk GD, Jaume JC, McLachlan SM, Rapoport B (1997). Engineering the human thyrotropin receptor ectodomain from a non-secreted form to a secreted, highly immunoreactive glycoprotein that neutralizes autoantibodies in Graves' patients' sera.. J Biol Chem.

[40] Chazenbalk GD, Wang Y, Guo J, Hutchison JS, Segal D (1999). A mouse monoclonal antibody to a thyrotropin receptor ectodomain variant provides insight into the exquisite antigenic conformational requirement, epitopes and in vivo concentration of human autoantibodies.. J Clin Endocrinol Metab.

[41] Churchill GA, Doerge RW (1994). Empirical threshold values for quantitative trait mapping.. Genetics.

[42] Williams RW, Strom RC, Goldowitz D (1998). Natural variation in neuron number in mice is linked to a major quantitative trait locus on Chr 11.. J Neurosci.

